# A streamlined multidisciplinary metabolic clinic in psychiatric recovery service: a pilot study

**DOI:** 10.3389/fpsyt.2024.1344453

**Published:** 2024-02-20

**Authors:** Kelvin CY. Leung, Bianca Bakr, Cindy Chung, Mayuri Parmar, James Elhindi, Vlasios Brakoulias

**Affiliations:** ^1^ Recovery Service, Cumberland Hospital, WSLHD, Sydney, NSW, Australia; ^2^ Research and Education Network, WSLHD, Sydney, NSW, Australia; ^3^ Faculty of Medicine and Health, The University of Sydney, Sydney, NSW, Australia; ^4^ School of Medicine and Translational Health Research Institute, Western Sydney University, Sydney, NSW, Australia

**Keywords:** metabolic syndrome, mental health consumers, streamlined approach, multidisciplinary team, inpatient program

## Abstract

**Background:**

The metabolic syndrome (MetS) is a collection of risk factors for cardiovascular disease and type-2 diabetes, that includes central obesity, hypertension, hyperglycaemia and dyslipidaemia. An audit indicated inadequate MetS screening in an Australian psychiatric recovery service.

**Objectives:**

We aimed to improve MetS screening, identification and intervention by offering streamlined lifestyle education, clinical reviews and discharge planning. This pilot program prioritized holistic, culturally-sensitive, patient-centric, and trauma-informed approaches to enhance metabolic health outcomes.

**Methods:**

A Metabolic Clinic was piloted in two psychiatric rehabilitation cottages (n=35), which involved disciplines of dietetics, exercise physiology, diversional therapy, occupational therapy, peer workforce, social work, clinical psychology, pharmacy, nursing and medical. Another cottage (n=15) was assigned as the comparison and received standard care. A 12-week, 3-times-per-week lifestyle and behavioral program, called *MetFit*, was devised and offered to those identified at screening for the treatment cottages. Outcome measures were feasibility measures, the five metabolic parameters (waist circumference, blood pressure, fasting serum triglycerides, high-density lipoprotein, and glucose), functional measures, and a meal questionnaire.

**Results:**

The treatment cottages had qualitative advantages in screening and identifying MetS. Of four enrolled consumers in *MetFit*, an improvement of triglycerides (p=0.08), squats (p=0.02), and push-ups (p=0.07) was observed. Major challenges of enrolment included an overall lack of acknowledgment of its importance, poor motivation of consumers and resources limitation.

**Conclusions:**

The one-stop provision of groups, peer support and inpatient pathway with multidisciplinary team-integration was generally accepted by consumers and the MDT and has iteratively demonstrated the urgent need for consumer-centered physical care and a cultural shift to foster collaboration within a psychiatric service.

## 1 Introduction

Metabolic syndrome (MetS) is a globally well-established cluster of risk factors for developing cardiovascular disease and type 2 diabetes (1). The core components of MetS include central obesity, hypertension, hyperglycaemia, and dyslipidaemia. In Australia, it affects 19% to 29% of the adult population aged over 25 years (2).

Given these statistics, MetS is a public health concern of considerable magnitude. People with mental health conditions have been shown to have a high rate of MetS, largely contributing to their limited life expectancy and reduced quality of life. Notably, individuals with schizophrenia experience a 20% reduction in life expectancy, with physical illnesses accounting for 60% of the excess mortality (3). Unhealthy dietary habits, sedentary lifestyles, and the use of antipsychotic medications may contribute to the high risk of MetS in this population (4). Furthermore, in the second Australian National Survey of Psychosis, 53% of participants aged 18–64 years met the criteria for MetS, indicating a significantly higher prevalence in this population, with approximately one-quarter of them at a high risk of experiencing a cardiovascular event within the next five years (5).

In light of the high prevalence of MetS among mental health consumers and its detrimental impact on life expectancy and quality of life, it becomes crucial for mental health services to prioritize the improvement of physical health outcomes, aligning with the NSW Ministry of Health strategy 2021 (6). Locally in a cohort from a psychiatric recovery service located in an Australian tertiary hospital, an audit was conducted on March 22, 2021, revealing inadequate screening for MetS among consumer patients (7). This was based on standardized criteria from a joint statement between international health organization issued in 2009 (8). The different cut-off points for waist circumference specific to their ethnicities are relevant to Australia’s multicultural setting. Out of a total of 87 patients across the five units, the screening rates for the five metabolic parameters within the preceding three months were: 99% for blood pressure, 71% for serum high-density lipoproteins (HDL), 71% for serum triglycerides (TG), 69% for fasting plasma glucose, and 64% for waist circumference. These findings indicated the pressing need for improved screening, identification, and implicitly, intervention of MetS within the service.

Despite an increased awareness of metabolic and physical health among mental health consumers, limited trials have evaluated the efficacy of a lifestyle intervention program in this context. Hence, based on our needs analysis, we have designed a one-stop pathway involving screening and identification of MetS, together with an intervention program aimed at enhancing physical and metabolic health within our service.

This pilot study aims to i) describe and trial a streamlined multidisciplinary pathway in addressing screening, identification and intervention of MetS among mental health consumers within a psychiatric rehabilitation service; ii) explore the feasibility and acceptability of a pilot exercise and diet program; iii) contribute to a model of physical healthcare that could be replicated in other mental health services.

## 2 Materials and methods

### 2.1 Design, settings and participants

This is a prospective pilot study. The study received ethical approval from the local Human Research Ethics Committee (HREC reference number: 2020/ETH01538; SSA reference number: 2021/STE02380). It was conducted between November 2021 and February 2023. A needs analysis was completed with a literature review, extensive multidisciplinary team (MDT) engagement and meetings, and consultation with the local Endocrinology Department. An integrated, MDT-led Metabolic Clinic, with follow-up plan in the community was proposed and piloted, consisting of disciplines of dietetics, exercise physiology, diversional therapy, peer workforce, occupational therapy, social work, clinical psychology, pharmacy, nursing and medical.

The psychiatric recovery service has a total of five units, three of which are open units and are known as ‘the cottages’, and the other two are secure units. The period of stay for the consumers in the open units is between 6 and 12 months. Given the similar model of care across cottages A, B and C, this pilot study was only conducted in these open units to minimize other associated confounders at this stage. Screening for MetS was completed in all three cottages (cottages A, B and C; *N*=50). Identification of MetS and the option to be referred to an intervention program, called *MetFit*, were piloted in two cottages (A and B; *n*=35), whilst one cottage (C; *n*=15) was assigned as the ‘control’ group receiving standard care (see *standard care*).

During MDT engagement, results of the MetS screening audit were shared with team members of different disciplines working at the cottages. Peer support workers and people with lived experience of mental illness were invited and had participated to give valuable insights into the potential challenges and development of the clinic. Common goals were discussed which informed the aims of this study. This clinic was conceptualized as MDT-led as each discipline possessed specific expertise (see **Appendix 1** for the roles of each discipline in the context of managing metabolic health in mental health consumers) and collectively they provided a holistic, culturally-driven, patient-centered and trauma informed care model for metabolic health.

### 2.2 Components of the metabolic clinic

The three-pronged approach of the Metabolic Clinic was as follow (see **Appendix 2** *for flow diagram of the clinic*):

#### 2.2.1 Screening

As part of the admission process and the three monthly MetS screening, nursing staff filled in the ‘MH Metabolic Monitoring Form’ on electronic medical record (eMR) for consumers in all three cottages. Specifically, they measured and completed the fields of:

- Systolic and diastolic blood pressure measurements
- Waist circumference measurements

Nursing staff started a conversation about metabolic syndrome with consumers, using the cue card provided to the staff (see **Appendix 3**).

Medical officers ordered blood tests and updated them on the same ‘MH Metabolic Monitoring Form’, including:

- Fasting lipids (including HDL and TG)
- Fasting blood glucose

#### 2.2.2 Identification

According to the Joint Interim statement (8) of the international organizations, for a person to be defined as having MetS, they must have three of the following five factors:

1. Abdominal obesity, defined as a waist circumference (WC) ≥94 cm in men and ≥80 cm in women for Europids, Sub-Saharan Africans, Eastern Mediterranean and Middle East (Arab) populations (ethnicity group A); and ≥90 cm in men and ≥80 cm in women for South Asians, Chinese, Japanese, South and Central Americans (ethnicity group B);
2. Serum triglycerides (TG) ≥150 mg/dL (1.7 mmol/L) or drug treatment for elevated triglycerides;
3. Serum high-density lipoprotein (HDL) cholesterol <40 mg/dL (1 mmol/L) in men and <50 mg/dL (1.3 mmol/L) in women or drug treatment for low HDL cholesterol;
4. Blood pressure ≥130/85 mmHg or drug treatment for elevated blood pressure;
5. Fasting plasma glucose ≥100 mg/dL (5.6 mmol/L) or drug treatment for elevated blood glucose.

In terms of selection criteria, for consumers, in cottage A and B, with the ‘MH Metabolic Monitoring Form’ completed, the MDT identified those at weekly meetings who had:

- metabolic syndrome (based on the criteria above; meeting 3 out of 5 criteria)
- risk towards metabolic syndrome (meeting 1-2 out of 5 criteria above)

Team invited all appropriate consumers who met the criteria of MetS and were at risk of MetS, to the intervention program, *MetFit*, as outlined below. Exclusion criteria include consumers i) undergoing acute phase of their mental illness; ii) potentially under the influence of their treatment regime, e.g. sedation; iii) having a physical impairment, affecting their ability to participate in exercise; and iv) who were non-English speaking.

#### 2.2.3 Intervention

##### 2.2.3.1 The *MetFit* program

The *MetFit* Program was a 12-week structured lifestyle and behavioral change program that encompassed evidence-based practices implemented by the *MetFit* team (see **Appendix 4** for the full program). The program aimed to educate mental health consumers about MetS and empower them to make behavior changes focusing on achieving long-term and sustainable healthy lifestyle habits. Participants were provided with practical opportunities to engage in group activities, including exercise sessions, leisure and recreation activities, nutritional education, and healthy meal preparation.

Group and individual nutrition consultations were conducted, which included an evaluation based on the Australian Guide to Healthy Eating (AGHE). The intervention comprised nutrition education in a group setting, incorporating modules on nutrition groups, healthy swaps, takeaway/snack options, and interactive sessions addressing topics such as sugar content in sugary drinks and reading food labels.

Exercise physiology sessions involved physical assessments to inform the prescription of safe and effective evidence-based exercise interventions. The questionnaires administered included the Physical Activity Stage of Change Assessment Tool and the Adult Pre-Exercise Screening Tool (APSS). Physical functional assessments encompassed the six-minute walk test (a sub-maximal exercise test assessing aerobic capacity and endurance), the 60-second push-up and sit-to-stand/squat test (evaluating upper and lower body muscular strength and endurance), and the single leg balance test (assessing static postural and balance control). The exercise program incorporated both aerobic, resistance and balance components. Participants attended 60-minute exercise sessions two times per week. During the supervised sessions, each participant received an individualized exercise program designed by the exercise physiologist which was tailored to their level of motivation, goals, and functional capacity. Individual workload, volume and progressive overload were monitored and recorded by the exercise physiologist. The type of resistance exercises involved a combination of machine-based, bodyweight and free-weight exercises. The aerobic component consisted of a dynamic stretching warm up followed by using either the treadmill, stationary bike, elliptical and/or the rower machine. Balance training using cones and hurdles as well as targeted exercises for pelvic floor strengthening were incorporated into the warm-up as per the needs of the group. The exercise physiologist provided education on the following areas: i) benefits of exercise on improving physical and mental health outcomes ii) instructions and feedback on proper form and safety considerations when exercising iii) physiological responses to exercise iv) long term adaptations to exercise training with emphasis on metabolic adaptations. Participants were also provided with handouts that reinforced this information for reference.

Diversional therapy utilized specialist skills in leisure theory, leisure behavior and activity adaptation. They provided, facilitated and coordinated leisure and recreational activities which were designed to support, challenge and enhance the psychological, spiritual, social, emotional and physical wellbeing of individuals who experience barriers to participation in the *MetFit* program thus improving their quality of life.

##### 2.2.3.2 Recruitment process and informed consent

The *MetFit* team consisted of disciplines of dietetics, exercise physiology and diversional therapy. The *MetFit* team implemented a comprehensive recruitment strategy. Emphasis was placed on effective communication and information dissemination to engage potential participants. Following the identification of consumers who met the criteria for the program, direct communication with the selected consumers commenced, providing them with an understanding of the program’s purpose and how it could support their pursuit of a healthy lifestyle.

To ensure maximum visibility, posters highlighting the program were strategically placed within the units and cottages. Identified consumers were invited to attend a recruitment/information session, which served as an opportunity to further explain the program, clarify expectations for participation, and assess their level of interest. Notably, the involvement of the peer workers played a pivotal role in this strategy, with their inclusion from the program’s inception fostering a sense of peer support and engagement (9).

Interested consumers in cottage A and B were approached again by the *MetFit* team. The *MetFit* team provided assurance that participants would receive comprehensive support throughout the program and that any pre-existing commitments would be accommodated. For instance, consumers receiving support services under the National Disability Insurance Scheme (NDIS) on weekdays were offered rescheduling options to ensure their participation. To streamline this process, the *MetFit* team coordinated closely with social workers to notify them of consumers’ involvement in the program and to facilitate necessary adjustments to their external support schedules. They were required to sign a consent form should they express their willingness to dedicate three times a week for a period of 12 weeks for the program.

##### 2.2.3.3 MDT integration

The dietitian, exercise physiologist, and diversional therapist offered specialist intervention and education to the participants at *MetFit*. The medical officers and pharmacist played a key role in reviewing and educating participants about necessary medications, including the risks and benefits of psychotropic medications, as well as prescribing antidiabetic, antihypertensive, and/or statin medications. Their collaboration and liaison with the Endocrinology Department ensured comprehensive medication management and guidance. Clinical psychologist, social workers and occupational therapists had ongoing roles to ensure participants’ psychosocial circumstances were optimal in adjusting to better motivation and healthy lifestyles. The occupational therapists co-facilitated exercise, leisure and recreation sessions with the *MetFit* team. (see **Appendix 1**)

Should participants be discharged from the cottages during the study period, those who were identified as having MetS, being at risk of MetS, and/or being invited to the *MetFit* program were communicated to their General Practitioners (GPs) and community services, including their case managers. This follow-up plan aimed to ensure continued monitoring and management of the participants’ metabolic health.

##### 2.2.3.4 Graduation ceremony

Sustaining 12 weeks of the *MetFit* program was an incredible accomplishment for consumers who previously had very little engagement in structured programs. A graduation ceremony was organised to celebrate their achievements.

Invitations were sent to the participants and a ‘plus one’ (family/carer/friend), peer workforce, nursing unit managers of the respective cottages, consultant psychiatrists and other medical officers, director of rehabilitation service, director of nursing, executive director and executive manager of mental health, and the *MetFit* Team (see **Appendix 5** for sample invitation). Invitations were emailed to the staff and hand delivered to the four consumers. Catering was organized for the invitees to enjoy a lunch after the ceremony. Funding was approved to use a local catering company for healthy options and individual lunch boxes, adhering to the covid safety guidelines. They consisted of a sandwich, fruits and a drink plus yogurt cups with fruit or granola toppings.

Certificates of completion were prepared for the *MetFit* participants (see **Appendix 6** for sample certificate). Additional certificates of individual achievements included Step Queen (for the most steps during *MetFit)*, Consistency Queen (for overall commitment to the *MetFit* program), Lift Queen (for lifting the heaviest weights), and Improvement Queen (for overall improvement).

Consumers were also gifted items as a thank you for their participation. The gifts were selected for consumers to continue to use in their healthy lifestyle journey and embodied our holistic approach. Items were those promoting self-care and wellbeing, including fitness equipment, a toiletry bag with deodorant, bodywash, sunscreen, lip balm, sheet mask, loofah, a healthy fitness journal and a pen. Director of Mental Health Services addressed the attendees. Speeches were given by the *MetFit* team with a lunch after the formalities.

##### 2.2.3.5 Confidentiality and privacy

Participants’ confidentiality was strictly maintained throughout. Information was only shared with their treating GPs, community services, and family/carers to enhance compliance with ongoing lifestyle and behavioral changes during and after discharge from the cottages. Respecting participant privacy and ensuring their autonomy over the sharing of information were fundamental principles of the clinic.

### 2.3 Standard care

Standard care involved referrals to the dietitian, exercise physiologist, or/and diversional therapist for individual support based on standard clinical guidelines. For the purpose of this data collection, cottage C was selected as the control group. Screening was done for all the consumers in cottage A, B and C. However, no active identification of MetS and referral option to *MetFit* program was available in cottage C, unlike in cottage A and B.

### 2.4 Outcome measures

#### 2.4.1 Feasibility measures

To explore the feasibility and acceptability of the Metabolic Clinic, a range of measures were employed.

Consumers’ attitudes and acceptability were gauged throughout the Metabolic Clinic. Following weekly MDT meetings, the number of participants who were identified from screening was recorded, together with the number of participants flagged by the MDT and invited to an information session. The *MetFit* attendance rate was monitored for program adherence. Observations, exercise behaviors, level of engagement and self-reported dietary changes were recorded through dietary logs and qualitative interviews, allowing for an evaluation of lifestyle changes and the program’s acceptability among participants.

The study formally collected feedback, discussions and qualitative insights regarding the experiences and perceptions of staff and clinicians involved at the weekly MDT meetings. This feedback involved their satisfaction with the program’s care, level of engagement, and motivation of participants, which were then iteratively used to refine the program approaches.

*MetFit* participants were asked to complete an evaluation form in their last sessions (*see* **Appendix 7**). They also provided valuable qualitative feedback at the evaluation session and at the graduation ceremony.

#### 2.4.2 Metabolic parameters

Data on the five metabolic parameters were collected at screening from all the three cottages. To assess potential impact of this pilot Metabolic Clinic, baseline data (pre-intervention data) was recorded three months after the first screening, and then another set of data three months after the baseline measure (post-intervention data) from all three cottages, whether they participated in *MetFit* or not.

#### 2.4.3 Diet questionnaire

For *Metfit* participants, the dietitian developed a nutrition knowledge questionnaire to ascertain food literacy levels and food choice behaviors. The questionnaire consisted of 14 closed questions (tick box options) and one open ended question. There were four knowledge-specific questions regarding the food sources and nutrition and ten self-reported questions regarding food intake habits and health consciousness. The questionnaire was administered in the first and last week of the 12-week program to assess the effectiveness of nutrition education and practical interventions on developing nutritional knowledge and influencing dietary habits and behaviors.

#### 2.4.4 Functional testing

For *MetFit* participants, the functional testing included the assessment of aerobic endurance, muscular strength and static and dynamic balance. Aerobic endurance was measured using the 6-minute walk test (6MWT) which is a self-paced, submaximal test of aerobic capacity. The participants were assessed on the distance walked over a span of 6-minutes. Upper body strength was measured using a maximum push up test. Participants were asked to perform push-ups against a wall and the maximum number of push-ups completed in 60-seconds was recorded. Lower limb strength was measured using a maximum squat test. Participants were asked to perform bodyweight squats and the maximum number of squats completed in 60-seconds was recorded. Balance was assessed using the tandem stance, tandem walk and single leg balance test (SLB left leg and SLB right leg).

Step-count monitors in the form of pedometers were utilized by participants during their weekly diversional therapy sessions. The participants attached the motion sensors to their waist band which objectively measured step-counts and served as a motivational means to encourage more movement and physical activity. Data from the pedometers was recorded at the conclusion of each session.

### 2.5 Statistical analyses

Acknowledging that our study primarily concerns acceptability and feasibility, the potential impacts of the Metabolic Clinic rolled out in cottage A and B versus standard care in cottage C on the metabolic parameters were explored. Baseline characteristics were compared using Chi-squared tests for categorical variables and t-tests for continuous variables. A series of paired-sample t-tests were performed to assess the magnitude and significance of changes in the five metabolic parameters before and three months after the baseline measurements. For those enrolled into the *MetFit* program (the intervention), in addition to the five metabolic parameters, functional measures (squats, push up, 6MWT, SLB left, SLB right; see *Outcome measures* above) were also examined for their significance. Hypotheses were conducted at a significance level of less than 0.05. All alternative hypotheses were two-sided. Analyses were conducted using R Studio Version 4.

## 3 Results

### 3.1 Feasibility outcomes

#### 3.1.1 Consumers’ attitudes and acceptability

The integration of streamlined screening, identification, and intervention within the psychiatric recovery service has yielded considerable interest and discussion among consumers at the cottages.

Positive feedback gathered during MDT meetings, especially from nursing unit managers, has indicated widespread acceptance of the three-monthly screening approach. Consumers, initially curious about the purpose of blood tests and waist measurements, engaged in conversations prompted by brief psychoeducation sessions with the nursing staff, fostering a discussion about MetS and spreading the discussion among consumers in the cottages. This encouraged enquiries about their MetS status, associated risks, and potential pharmacological options at their medical reviews.

The feasibility of MetS identification and referral for *MetFit* were reported to be inclusive by adopting an MDT approach, as consumers had individual needs, such as considering fluctuations in their mental states and scheduling sessions during medication-sensitive times and support worker visits. However, the *MetFit* team was unable to accommodate individual requests for a rescheduled program due to *MetFit* being a group intervention. With these considerations in mind, the pre-intervention screening identified 22 out of 35 consumers meeting the criteria of MetS; while 7 were identified to be at risk of MetS. The MDT flagged 16 consumers to be suitable for the *MetFit* program and they were invited to an information session.

Following the recruitment/information session, an initial cohort of eight consumers expressed their intention to participate in *MetFit*. The cottages were notified of their involvement, and staff received instructions on how they could provide support during the program. Prior to the program’s commencement, staff members were requested to assist consumers in acquiring appropriate clothing and footwear for the exercise activities. Additionally, they were encouraged to remind consumers of the scheduled sessions and provide motivation for regular attendance.

Half of the initially interested consumers chose not to proceed. While one consumer expressed concerns about the program’s duration and perceived difficulty, another attended only the initial weeks before discontinuing. One consumer was reluctant to change their designated days for external support, resulting in the clash of their scheduled activities. Their motivation for engaging in the program emerged as one of the key factors. Furthermore, one consumer sustained a foot injury not related to the program halfway through and therefore had to terminate.

A final sample size of four consumers successfully completed the entire 12-week program. It is worth noting that they were female and of ethnicity group A, with age ranging from 33 to 47.

The participants provided ongoing feedback throughout the *MetFit* program. This qualitative feedback that was captured during the intervention was clinically significant. The participants regularly remarked how they felt better after the physical activity sessions. They felt motivated seeing their step count increase each week and felt stronger as they increased their repetitions when using the weights and increased time on the treadmill and exercise bike. Allowing consumers to choose the types of exercise and recreational activity improved their sense of health ownership, participation and engagement. During the *MetFit* program, they particularly enjoyed the exercise circuits and boxing sessions as the group setting helped to boost their self-esteem and improve their social connections with one another. Participants were observed to positively encourage, motivate and compliment their peers. The weekly incentives and smoothies received a positive response as it contributed to their rewards and motivation and provided the participants with an opportunity to implement the theoretical skills they had learnt in a practical setting. Overall, the intervention fostered an increased awareness of good physical health and adopting positive lifestyle changes. The MDT worked together to motivate and encourage participants during the 12 weeks.

Participants’ enthusiasm for adopting a more active lifestyle increased as a result of being involved in the intervention. A change in behavior was evident when participants were punctual to exercise sessions two days per week and did not require to be followed up by staff facilitators on the day. Two participants were observed to wait patiently outside the gymnasium each week in preparation for the sessions to commence. Participants were receptive to constructive feedback on their form when performing exercises and adjusted their technique in accordance with the feedback they received. They demonstrated an increased body awareness and actively sought feedback from the Exercise Physiologist on whether they were performing their exercises safely. Additionally, participants were provided with pain education and in turn, developed a good understanding of pain management and appropriate behaviors in response to pain. Prior to the commencement of each exercise session, participants notified the Exercise Physiologist of any new areas of pain or discomfort. Where appropriate, the Exercise Physiologist prescribed the participant with modified exercises and specific exercises to help them self-manage.

At the graduation ceremony, one consumer provided a handwritten thank you note (which they planned on reading at the graduation ceremony) however decided to give it to the clinician to read. They thanked the team for supporting her to be healthy and believing in her. They were also proud to feel better, to have lost weight and to have their clothes fitting comfortably. Participants looked forward to group sessions and discussed healthy habits with her cottage peers. Staff and Executive Team offered congratulations and praise for their dedication and motivation over the 12 weeks.

#### 3.1.2 Staff feedback

Having cue cards available for staff facilitated conversations about metabolic health, aiding in the initial interest among the consumers within the psychiatric recovery service. Collaboration with the Endocrinology Department confirmed that the *MetFit* Program aligned with current evidence-based guidelines, emphasizing its structured approach and focus on fostering health promotion while monitoring and enhancing metabolic outcomes.

Most consumers were already known to the *MetFit* clinicians hence it was easier to establish rapport during the program. However, operational challenges in obtaining blood samples and accessing scales for weighing consumers could be impacted by scales being used or taken away from the consult room.

During week 1, some resistance in completing baseline functional measures among participants was observed. One participant declined the 6-minute walk test, while another felt self-conscious and initially refused the single leg balance assessment. Acknowledging their discomfort, the clinicians provided reassurance and assessments in adjacent room where they felt more comfortable.

The enthusiasm and interest shown by the Executive team, nursing unit managers, and allied health staff were keen to know if the program would be rolled out to other units and cottages. The observable positive impact and flow-on effects to other consumers within the service might imply potential future expansions of the program.

#### 3.1.3 *MetFit* evaluation forms

The four *MetFit* participants rated each aspect of the program 5 out of 5. They agreed that the program significantly increase their understanding of exercise and healthy eating, in turn improving their confidence in managing their health and physical wellbeing. Overall, they collectively rated the program as ‘excellent’. In the final evaluation session, participants unanimously praised its supportive environment and comprehensive approach.

### 3.2 Metabolic parameters comparing between the Metabolic Clinic and control

Waist measurement, diastolic blood pressure, glucose, TG, and HDL at baseline were comparable between the Metabolic Clinic (cottage A and B; n=35) and control (cottage C; n=15) (**Table 1**). Systolic blood pressure, however, was marginally greater in the clinic (p=0.05; 128.69 mmHg, SD 12.71 in the clinic versus 119.36 mmHg, SD 15.00 in control). At follow up, all metabolic parameters were comparable between the Metabolic Clinic and control.

**Table 1:** Differences in the characteristics between treatment and control cottages.

Characteristic	Treatment with the Metabolic Clinic: Cottages A and B (n = 35)	Control: Cottage C (n = 15)	P value
Demographic Features^#^			
Sex: Male	42.86% (15)	53.33% (8)	0.55
Sex: Female	57.14% (20)	46.67% (7)	
Ethnicity Group A*	77.14% (27)	86.67% (13)	0.70
Ethnicity Group B**	22.86% (8)	13.33% (2)	
Baseline Metabolic Measurements^$^			
Waist circumference (cm)	112.33 (18.90)	111.40 (29.97)	0.91
Systolic blood pressure (mmHg)	128.69 (12.71)	119.36 (15.00)	0.05
Diastolic blood pressure (mmHg)	79.60 (13.99)	78.55 (8.58)	0.82
Glucose (mmol/L)	6.51 (2.57)	5.78 (1.23)	0.35
Triglycerides (mmol/L)	1.85 (0.90)	1.98 (0.88)	0.67
High-density lipoprotein (mmol/L)	1.11 (0.31)	0.99 (0.21)	0.24
Follow-Up (12 weeks after baseline) Metabolic Measurements^$^			
Waist circumference (cm)	106.04 (23.22)	112.75 (15.17)	0.38
Systolic blood pressure (mmHg)	118.68 (12.41)	117.08 (10.15)	0.70
Diastolic blood pressure (mmHg)	76.73 (7.11)	72.77 (10.24)	0.19
Glucose (mmol/L)	5.91 (1.46)	5.71 (1.18)	0.66
Triglycerides (mmol/L)	2.02 (0.95)	2.26 (0.94)	0.46
High-density lipoprotein (mmol/L)	1.01 (0.26)	1.34 (1.28)	0.24

^#^reporting proportion (n).

*A: Europids, Sub-Saharan Africans, Eastern Mediterranean and Middle East (Arab) populations.

**B: South Asians, Chinese, Japanese, South and Central Americans.

^$^reporting mean (standard deviation).

In the Metabolic Clinic, there was a decrease in the paired systolic blood pressure from the beginning to end of the study period (p=0.05; mean difference -6.45 mmHg, SD 14.26) and a significant decrease in the paired mean HDL (p=0.01; mean difference -0.10 mmol/L, SD 0.15). In the control cottage, we did not observe any significant difference in the paired metabolic parameters. Additionally, no other metabolic parameter had a significant difference (**Table 2**).

**Table 2:** Paired metabolic measurements* by treatment and control cottages.

	Treatment Cottages with the Metabolic Clinic: Cottages A and B				Control Cottage: Cottage C				Diff.
Measurement	Pre	Post	Diff	p	Pre	Post	Diff	p	
Waist (cm)	112.33 (18.90)	106.04 (23.22)	-2.85 (15.84)	0.41	111.40 (29.97)	112.75 (15.17)	2.22 (24.05)	0.79	0.36
Systolic blood pressure (mmHg)	128.69 (12.71)	118.68 (12.41)	-6.45 (14.26)	0.05	119.36 (15.00)	117.08 (10.15)	-4.20 (16.23)	0.43	0.24
Diastolic blood pressure (mmHg)	79.60 (13.99)	76.73 (7.11)	-1.41 (15.42)	0.67	78.55 (8.58)	72.77 (10.24)	-5.20 (9.90)	0.13	0.55
Glucose (mmol/L)	6.51 (2.57)	5.91 (1.46)	-0.53 (2.67)	0.36	5.78 (1.23)	5.71 (1.18)	0.07 (0.95)	0.81	0.40
Triglycerides (mmol/L)	1.85 (0.90)	2.02 (0.95)	0.04 (0.48)	0.73	1.98 (0.88)	2.26 (0.94)	0.38 (0.88)	0.17	0.23
High-density lipoprotein (mmol/L)	1.11 (0.31)	1.01 (0.26)	-0.10 (0.15)	0.01	0.99 (0.21)	1.34 (1.28)	0.35 (1.44)	0.42	0.12

*reporting mean (standard deviation).

### 3.3 Metabolic parameters of *MetFit* participants

The four participants enrolled in *MetFit* observed no statistically significant change in any of the metabolic parameters from beginning to end (**Table 3**). A mean decrease in waist measurement of 3.78 cm (p=0.10; SD 3.16) is noted. A mean decrease of 0.23 mmol/L (p=0.08; SD 0.17) was also observed for triglycerides.

**Table 3:** Characteristics before and after the Metabolic Clinic and the 12-week *MetFit* in N = 4 participants*.

Characteristic^#^	Pre	Post	Difference	P value
Waist (cm)	129.40 (35.14)	125.63 (34.92)	-3.78 (3.16)	0.10
Systolic blood pressure (mmHg)	125.75 (6.18)	119.25 (12.84)	-6.50 (16.66)	0.49
Diastolic blood pressure (mmHg)	83.50 (5.57)	76.25 (7.93)	-7.25 (10.97)	0.28
Glucose (mmol/L)	7.95 (6.17)	5.25 (0.91)	-2.70 (5.27)	0.38
Triglycerides (mmol/L)	2.15 (0.75)	1.93 (0.65)	-0.23 (0.17)	0.08
High-density lipoprotein (mmol/L)	1.03 (0.34)	1.00 (0.37)	-0.03 (0.10)	0.64
Squat	20.75 (4.86)	39.50 (7.55)	18.75 (7.63)	0.02
Push Up	20.00 (5.48)	35.25 (7.41)	15.25 (10.81)	0.07
6MWT (m)	304.67 (25.82)	287.75 (63.10)	1.00 (37.81)	0.97
SLB Left	5.00 (4.36)	13.13 (16.05)	11.00 (14.00)	0.31
SLB Right	4.00 (3.12)	12.00 (12.03)	8.00 (11.69)	0.36
Pedometer (steps)	114.33 (22.12)	937.33 (898.79)	823.00 (882.25)	0.25

*Three participants met the criteria for metabolic syndrome (MetS) at baseline, while one did not. After 3 months, the MetS status of each participant remained the same. It should further be noted that all 4 participants were female and of ethnicity group A.

^#^reporting mean (standard deviation).

Three out of four participants met the criteria for metabolic syndrome at baseline. After 12 weeks, the metabolic syndrome status of each participant remained the same.

### 3.4 Diet questionnaire of *MetFit* participants

There were improvements across the nutritional knowledge and food intake habit sections. Participants were able to correctly identify food sources related to nutrients such as healthy fats and those with high sugar content. Self-reported food intake habits improved and were closely aligned with the Australian Guide to Healthy Eating (AGHE) across all domains including vegetable, fruit and water intake. While there were increase in meal frequency and no change in consumption of sugar sweetened drinks and takeaway meals, there were improvements in health consciousness and quality of eating habits.

### 3.5 Functional outcome measures of *MetFit* participants

There were statistically significant improvements in participants’ maximal 60-second squat (p=0.02; mean increase 18.75, SD 7.63) and push up (p=0.07; mean increase 15.25, SD 10.81) tests (**Table 3**). Aerobic fitness measures varied between participants and the mean change was not statistically significant for any measure.

Narratively, there were improvements in maximum strength testing for the upper and lower limbs. All participants increased their output from baseline in both the maximal 60-second squat and push up tests. In terms of aerobic fitness, one participant increased their 6-minute walk distance by 54-meters which was considered to be clinically significant. All participants achieved improvements from baseline in their single-leg balance bilaterally.

## 4 Discussion

As far as the authors are aware, the implementation of an MDT-led Metabolic Clinic with an intervention component in an inpatient psychiatric unit has not been previously explored. We described our unique approaches in this study. This service improvement initiative also received overall positive staff and consumers’ acceptability and fostered encouraging attitude towards physical health care within our psychiatric recovery service. Albeit being a pilot study and the operational difficulties as discussed above, it has shown that implementing a streamline approach to screening, identification and intervention of MetS in mental health rehabilitation units was preferred by the MDT and the consumers alike. We acknowledged the final small sample size of participants in *MetFit* (the intervention). Although our pilot study only showed limited statistical significance in some parameters of metabolic and functional measures, our findings may indicate a real-life benefit to participants, who reported to be moving more, eating healthier and feeling better as evident by the functional and qualitative outcomes. Nevertheless, the high prevalence of MetS in our pilot study cohort, also noted in the existing literatures (3, 5, 10), has emphasized the importance of addressing this condition among mental health consumers.

The emphasis of an MDT design in our Metabolic Clinic specifically contributed to its comprehensive, holistic, and collaborative care across the service. Vreeland (11) has highlighted that people with serious mental illnesses fall through the gaps in the healthcare system. This is often exacerbated by barriers to accessing quality physical care, lifestyle interventions and early detection of metabolic risks. He suggested a model where every individual’s health status is consistently assessed, with monitoring protocols established for those on antipsychotic medications and seamless communication between behavioral health and primary care providers. Other studies have also supported the importance of interdisciplinary collaboration in addressing the complex health needs of psychiatric patients (12, 13). The MDT-led nature of our clinic could hence promote a culture of continuous improvement within the recovery service and a sense of empowerment both for the consumers and interprofessionally.

The role of the peer workers helped frame the clinic as a personal journal towards their overall wellness. Using their lived experiences, peer workers are uniquely positioned to build genuine connections, inspire hope, and model recovery (14). Moreover, their influence was evident in the recruitment phase, where they offered authentic and relatable dialogues with potential participants. Drawing from the experience of a community-based rehabilitation service (15, 16), an expansion of their role might involve more frequent check-ins during metabolic interventions to mitigate feelings of the program being overwhelming and foster trust-building. Additionally, peer workers could enhance staff empowerment and collaboration during their involvement in regular MDT debriefing and training sessions. However, Gillard and Holley (17) summarized the potential challenges, highlighting the complexities when the role of peer workers becomes indistinct, misaligned with managerial perspectives, or when there is insufficient organizational support for consumer recovery.

Some unique features of our Metabolic Clinic were the provision of group sessions and the integration of an inpatient one-stop design, facilitating coordination among different healthcare providers and streamlining the pathway for metabolic care. Group-based interventions have shown promise in improving lifestyle behaviors and weight-loss programs in other studies, owning to inter- and intra-personal processes within group dynamics (18). Further, an inpatient integration of the Metabolic Clinic potentially allows for resource optimization. Specifically in our rehabilitation setting, there can be a considerable wait time to coordinate the community psychosocial needs of consumers before they can be discharged. Although not specifically shown in our pilot study, inpatient-initiated interventions could foster consumers’ sustainable interest of healthy lifestyle in the community, confirmed by qualitative data from other inpatient programs such as the Keeping the Body in Mind program in a youth service (19). When considering our clinic model’s transferability to other acute inpatient services, a 12-week long inpatient program might not be feasible when the admission and discharge turnover is significantly higher. Communication with community services and timely referrals to appropriate lifestyle interventions that address MetS might instead be the focus in such a case.

Recognizing the challenges presented by a cohort of consumers with chronic mental health conditions in a psychiatric rehabilitation setting, it is important to highlight the impact of negative symptoms, sedation and psychopathological manifestations on their engagement in our Metabolic Clinic. The observed low participation rates in the program were suggested to be related to these factors, seconded by a systematic review and meta-analysis regarding the barriers towards exercises in severe mental illness (20). The review emphasized the necessity for professional support in improving motivation, aligning with our initial strategy to actively involve psychologists, pharmacists, and medical officers in conducting motivational consultations and providing lifestyle education even before the recruitment phase for *MetFit*. This further indicates the need in adopting an MDT approach to minimize motivational barriers and encourage greater participation.

Based on our pilot study, insights for future research and implementation have been shed. Firstly, while our Metabolic Clinic has demonstrated partial feasibility, a deeper evaluation with more participants and a larger sample size would be warranted, as currently we acknowledge that the data represent the averages obtained in groups rather than that of an individual participant, increasing the risk of a Type 1 error. Secondly, the iterations of the clinic and longer-term health outcomes (i.e. functional and metabolic markers) would be useful to determine its clinical significance. Thirdly, collecting systematic qualitative data on participants’ perspectives on the clinic via formal qualitative methodologies would provide valuable information for the clinic’s refinement and optimization. Lastly, although the limited sample size of our pilot study has limited the generalizability of our findings, the identified participatory challenges offer key insights for the next steps. As noted above, challenges such as perceived program length, fostering motivation throughout *MetFit*, coordinating external support services during the 12-week program, and streamlining communication across participating cottages have implied room for improvement (21).

Our pilot study in establishing a unique streamline approach to metabolic health in the recovery service has provided some insights in providing specific, fit-for-purpose, motivational care to this cohort of consumers living with mental illness. The collaborative and consumer-centered approach of the clinic, combined with its MDT-led design and integration with existing services, showcases the potential for sustainable care delivery. The ongoing commitment to consumer-centered physical care and the culture of collaboration fostered by the clinic could serve as guiding principles for the future development and incorporation of physical health care in psychiatric services.

## Data availability statement

The raw data supporting the conclusions of this article will be made available by the authors, without undue reservation.

## Ethics statement

The study involving humans was approved by Western Sydney Local Health District Human Research Ethics Committee. The study was conducted in accordance with the local legislation and institutional requirements. The participants provided their written informed consent to participate in this study.

## Author contributions

KL: Conceptualization, Data curation, Formal analysis, Funding acquisition, Investigation, Methodology, Project administration, Resources, Software, Supervision, Validation, Visualization, Writing – original draft, Writing – review & editing. BB: Writing – review & editing. CC: Writing – review & editing. MP: Writing – review & editing. JE: Formal analysis, Writing – review & editing. VB: Supervision, Writing – review & editing.
