# Supplementary material for: A streamlined multidisciplinary metabolic clinic in psychiatric recovery service: a pilot study
Source: Front Psychiatry. 2024 Feb 20;15:1344453. doi: 10.3389/fpsyt.2024.1344453 (PMC10913053; doi:10.3389/fpsyt.2024.1344453)
Supplement: Supplementary file 1 [file DataSheet_1.pdf]

MetFit Graduation

The MetFit team invite you to celebrate the graduation of the MetFit participants

Date: Wednesday 14th Dec 2022

Time: 11:00 am

Venue: ___________

RSVP: Friday 9th Dec 2022

**RSVP essential as this is a catered event. Please note your dietary requirements in the meeting invite*
