# Supplementary material for: A streamlined multidisciplinary metabolic clinic in psychiatric recovery service: a pilot study
Source: Front Psychiatry. 2024 Feb 20;15:1344453. doi: 10.3389/fpsyt.2024.1344453 (PMC10913053; doi:10.3389/fpsyt.2024.1344453)
Supplement: Supplementary file 2 [file DataSheet_2.pdf]

CERTIFICATE

OF ACHIEVEMENT

This Certificate is Presented To :

____

For the completion of the
12 week MetFit program

PROJECT LEAD

DIETITIAN

DIVERSIONAL THERAPIST

EXERCISE PHYSIOLOGIST

14th December 2022
