# Supplementary material for: A streamlined multidisciplinary metabolic clinic in psychiatric recovery service: a pilot study
Source: Front Psychiatry. 2024 Feb 20;15:1344453. doi: 10.3389/fpsyt.2024.1344453 (PMC10913053; doi:10.3389/fpsyt.2024.1344453)
Supplement: Supplementary file 3 [file Table_1.docx]

Appendix 1. Multidisciplinary roles

**Dietitian**

Dietitians are qualified in nutrition and dietetics, and these qualifications are recognised by a national authority. The dietitian applies the science of nutrition to the feeding and education of individuals and groups of people in health or with specific disease including MetS^1^.

Whilst MetS may have a genetic component to its development, many of the symptoms are related to lifestyle factors such as diet and exercise^2^. Lifestyle changes as outlined in the Australian Dietary Guidelines are effective for the management of obesity, high blood pressure and elevated triglycerides^2^. A reduction in weight by 5-10% and increasing physical activity can significantly reduce a person’s risk of going on to develop Type 2 Diabetes Mellitus (T2DM) ^2^.

Evidence also suggests that the type of carbohydrate eaten may impact on the body’s insulin requirements. Low glycaemic index (GI) foods such as wholegrain breads and cereals, legumes, dairy foods and fruit are better dietary choices than high GI foods including white bread, biscuits and cake^2^. High GI foods require more insulin to effectively clear glucose from the bloodstream in order to maintain blood glucose levels within the normal range (4-7.8mmol/L) ^2&3^. Ongoing increased insulin requirements to manage a high GI diet places long term stress on the body^2^.

To reduce your risk of MetS one needs to^2&4^:

- Follow a healthy eating plan. Choose natural, fresh foods and limit processed foods. Use less salt, choose healthy fats, opt for low GI choices, and snack on high fibre foods like nuts & fresh fruit.
- Maintain a healthy body weight.
- Be physically active.
- Don’t smoke.

**Exercise Physiologist**

An Exercise Physiologist is a university qualified allied health professional with training to screen, assess and apply clinical reasoning to ensure patient safety and appropriateness of; evidence based individualised exercise interventions, provide health education and implement lifestyle modification and motivational strategies^1^.

The Mental Health Commission of NSW^2^ states that successful lifestyle interventions must be multifaceted with a focus on diet, physical activity and behavioural therapy that is tailored for each individual. Furthermore, studies have found that a combination of both group and individualised sessions for, socialisation, peer support and adherence, and one-on-one attention and individualised programming, respectively, had a greater effect compared with using either strategy in isolation^3,4^.

Literature states that a combination of both aerobic and resistance exercise is necessary for prevention and management of MetS risk factors^5^. Exercise has been shown to improve blood pressure, improve insulin sensitivity and glycaemic control, reduce waist circumference and overall body weight and improve cardiorespiratory fitness^6^. One of the greatest benefits of exercise includes significant reductions in abdominal fat independent of overall weight loss. This is a particularly important factor as abdominal obesity is related to metabolic dysfunction and cardiovascular disease, factors which contribute to MetS^,7^. There is current evidence for both moderate intensity cardiovascular and resistance training and higher intensity interval training (HITT) for management of MetS in individuals with Mental Illness^8^.

Exercise Physiology interventions include:

- Pre-exercise screening and SMART goal setting. Pre and post intervention measures of x2 questionnaires (SIMPAQ and DASS-21) and physical functional testing.
- Individualised moderate-to-vigorous intensity aerobic exercise and progressive resistance training programs. The programs will focus on large muscle groups and tailored at improving metabolic outcomes.
- Education on principles of safe exercise, physical activity vs. exercise, risks associated with sedentary behaviour and benefits of exercise for mental health and cardio-metabolic risk factors.

**Diversional Therapist**

Diversional Therapists implement behavioural, functional and educational changes through the use of leisure and recreation activities and programs. Individuals are supported to develop or improve meaning in many domains which include leisure participation, health behaviour change, personal resilience, improved functioning and developing an active lifestyle.

To ensure best practice Diversional Therapists have an understanding and implementation of the Leisure Ability Model and conduct leisure assessments using standardised measures such as Idyll Arbor Leisure Battery and other assessment tools to ascertain an individual’s skills, limitations, interests and previous leisure experiences.

In terms of what Diversional Therapists may be able to provide (pending resources, therapeutic environment and ongoing funding) could include individual leisure assessment, development of leisure focussed goals, leisure education sessions and opportunities to engage in meaningful leisure participation.

Measureable outcomes include social cohesion, health behaviour change, leisure participation, mood regulation, symptom reduction and improved functioning.

**Social Worker**

MetS can affect the family unit as a whole, not just the individual experiencing the psychotic episode. As the person’s body shape changes and physical health declines, their mental health may suffer further as a person may become isolative, anhedonic, irritable, lonely, and depressed. As such, the family may feel they are losing their loved one as they feel at a loss as to what to do to support them.

During the course of the affected person’s life, the family may see changes in behaviour, routines and social, vocational and educational interactions. Family members may find it easier to appease their loved one rather than encourage them to change behaviours positively, albeit all caring their intentions are. This is an area that education can improve to help motivate positive change for the health and wellbeing of the person. Not only this, the person could also be experiencing devastating effects of medications which could have impacts on sexual activity, thus inability to build or sustain relationships. If they are already a family man/woman, they may be experiencing the inability to actively participate in the role of a caregiver, thus feeling a sense of loss of role within the family unit. If they had previously been working, the illness took hold and as a result have lost their job, there is another sense of loss which could further lead to low self-esteem and low confidence. **Social Workers** support family with some of the aforementioned concerns through education, improving relationships and referral pathways.

A number of research suggests that improving certain factors such as one’s coping capacities, perception of supports received, and access to education can reduce the impact on the individual as well as the family. Thus for those diagnosed with or at risk of MetS, integrating family and carers support can provide similar benefits not only from reducing psychological distress but also coping with MetS and learning how to reduce and/or manage the risk of MetS. Research suggests that this could be achieved by attending family and carer groups or within the family network.

In addition, family can support their loved ones by encouraging the person to continue to see their GP, keeping routines, giving them space and involving family as part of their normal practice. Diet, exercise and smoking cessation can lead to better sleep pattern, motivation, self-esteem, confidence and general wellbeing. Modifying the lifestyle and discussing medications changes/regimes with the person’s GP/Psychiatrist can all benefit towards reducing the prevalence and risk factors of developing MetS.

**Clinical Psychologist**

There is considerable evidence for the effectiveness of health psychology interventions in both the prevention and treatment of lifestyle-related chronic diseases. The effectiveness of such interventions lies in their focus on addressing life-style changes. In the context of the MetS these primarily comprise weight loss, diet and exercise.

MetS is associated with increased risk for both diabetes and cardiovascular disease. Psychological interventions have been shown to be effective in the prevention of each through behavioural interventions that address life-style changes. Psychological interventions have also been found to be effective in the treatment of each, for example, in reducing anxiety and depression for patients with chronic heart disease and in symptom reduction for individuals with diabetes.

Specifically, interventions such as motivational interviewing and behavioural modification have been found to significantly improve risk factors associated with MetS. Motivational interviewing works with a patient's low motivation to change by eliciting the patient's own values, reasons and goals for change. Behaviour modification reduces maladaptive behaviours through the use of positive reinforcement techniques.

Using interventions such as psychoeducation, behavioural activation, motivational interviewing and behaviour modification, psychology has a unique role to play in addressing factors related to the prevention and treatment of MetS across disciplines within the inpatient setting. In terms of prevention, psychological interventions have the potential to assist in improving life-style choices in areas such as exercise and diet, while in terms of treatment, psychology may also assist in areas including medication compliance.

**Occupational Therapist**

Research indicated that Occupational Therapy intervention is important in reducing the risk of MetS. This is as a result of many Occupational Therapy clinicians commonly encounter clients diagnosed with or who are at risk of MetS across all populations and setting. Through the intensive research it has highlights that MetS affects participation in occupation, and therefore occupational therapy intervention can assist with identifying and developing a program to reduce and prevent risk factors. Occupational Therapy focus on lifestyle interventions and improving motivation to take control of their own life. Therefore providing the clients with the skills to empower themselves and make positive Meaningful and healthy lifestyle.

The primary role of an occupational therapist would be to assist with the effects of MetS. This includes providing adaptations for occupational challenged caused by MetS.

Occupational Therapy intervention include:

- Apply to any client population
- Create and promote prevention and identification of risk factors
- Establish routine
- Restore (remediation and restoration of function despite the MetS
- Maintain a healthy lifestyle
- Modify occupational function
- Prevention of MetS

Additional intervention that would reduce the risk of MetS includes incorporation or family and carers into the care plan of the clients. Having a client’s social supports included in the care, decreases risk of MetS. The interventions would include increases motivation, family and carer groups, and developing skills to improve a structured routine.

**Pharmacist**

As part of the MetS Project, Cumberland Pharmacy will conduct a thorough medication review in Waratah and Banksia to assess cardiometabolic risk based on:

- Medications and medication combinations associated with increased body weight
- Antipsychotic medications associated with high risk of cardiometabolic adverse effects mainly *clozapine*, *olanzapine* and *quetiapine*.^1,2^
- Antidiabetic medications associated with weight gain (Sulfonylureas versus GLP-1 agonists).

Pharmacological interventions, upon liaison and collaboration with medical practitioners, may include,

- Switching to a different medication that is less likely to cause cardiometabolic adverse effects. However, medication history and previous unsuccessful trials of other medications may support the continuation of the current treatment regimen.

(Note: Within the range of doses commonly used for schizophrenia, there is no evidence of a dose relationship for metabolic disorders associated with clozapine. Dose reduction is therefore not a strategy to mitigate or manage the cardiometabolic risk. ^3^)

- Adding metformin or aripiprazole (5 -15mg)^3,4^ to the treatment regimen to control weight gain and associated insulin resistance.

(Note: Renal function is to be checked before introducing metformin; Weight loss has been observed when *aripiprazole* was added to clozapine and to olanzapine. Not recommended with other antipsychotics.^3,4^)

- Adding or adjusting the antidiabetic medications to control wight gain caused by some antidiabetic medications (sulfonylureas and insulins) and from the other side to promote weight loss (GLP-1 agonists) and more cardiac and renal protective effects (SGLT2 inhibitors).^5^
- Adding or adjusting statin/fibrate doses based on the assessed risk.^6^

*Pharmacy will also:*

- Organise and provide pharmacy education to consumers about medications and possible side effects. This will be done in collaboration with other members of the Allied Health team to encourage and support lifestyle modifications including healthy eating and physical activity.
- Measure the change in the cardiometabolic risk following intervention (the period of the study).

**Nursing Staff**

Ward and cottage staff work very closely with the patients. They play an essential key role as follow:

- Initial Metabolic screening, including blood pressure & waist measurement.
- Recording blood results on the Metabolic Monitoring form
- Initial & ongoing conversation about MetS which includes educating the clients and their carers, answering their questions, making them aware of the Metabolic Clinic.
- Monitoring and reminding the doctors about:
  1. Ordering bloods and repeat ordering when we receive abnormal results.
  2. Attending to physical exam during handover.
- Monitoring and documenting our client’s:
  1. Diet intake
  2. Activity in the unit.
  3. Medication usage including regular and PRNs.
  4. Compliance to intervention and barriers to compliance.
- Referrals to dietitian, exercise therapist and social worker for specific needs.
- Handing over required tasks related to metabolic screening during handover.
- Participating in quality improvement and governance for any improvements and changes in service delivery.

**Medical Practitioner**

Psychiatric patients have a greater risk of premature mortality, predominantly due to cardiovascular diseases (CVDs). Convincing evidence shows that psychiatric conditions are characterized by an increased risk of MetS, a clustering of cardiovascular risk factors including dyslipidaemia, abdominal obesity, hypertension, and hyperglycaemia. This increased risk is present for a range of psychiatric conditions, including major depressive disorder (MDD), bipolar disorder (BD), schizophrenia, anxiety disorder, attention-deﬁcit/hyperactivity disorder (ADHD), and post-traumatic stress disorder (PTSD).

Medical practitioners play a role in screening, identification and intervention of MetS. As individuals with mental illnesses are more vulnerable to develop MetS, a regular physical examination will help in early identification. Medical practitioners will measure blood pressure and do a complete blood work that may indicate the early development of MetS.

Early detection, identification of high-risk patients, and early treatment based on existing guidelines will be part of their daily practice. Another obvious route to prevent or improve the risk of MetS is by directly modifying the lifestyle of psychiatric patients.

These patients would also be psychoeducated about their mental illness, their medications and the increased risk of developing complications. As part of the intervention, mental health clinicians will review medications and also screen these patients for any medical comorbidities that might further increase their risk for MetS.

**References**

**Dietitian References**:

1. Dietitians Association of Australia website, *Definition of a Dietitian*, <https://daa.asn.au/what-dietitans-do/definition-of-a-dietitian/>, 2020.
2. Dietitians Association of Australia website, *What is Metabolic Syndrome?* <https://daa.asn.au/smart-eating-for-you/smart-eating-fast-facts/medical/what-is-metabolic-syndrome/>, 2020.
3. Diabetes Australia website, *Blood Glucose Monitoring,* <https://www.diabetesaustralia.com.au/blood-glucose-monitoring>.
4. Australian Healthy Food Guide, *Metabolic Syndrome: Beat the silent killer*, April 2018.

**Exercise Physiologist References**:

1. Accredited Exercise Physiologist Scope of Practice: https://www.essa.org.au/Public/Consumer_Information/What_is_an_Accredited_Exercise_Physiologist_.aspx 2018
2. Mental Health Commission of NSW (2016). Physical health and mental wellbeing: evidence guide, Sydney, Mental Health Commission of NSW
3. Ward MC, White DT, Druss BG. A meta-review of lifestyle interventions for cardiovascular risk factors in the general medical population: lessons for individuals with serious mental illness. The Journal of Clinical Psychiatry. 2015;76(4):e477-486.
4. Bruins J, Jörg F, Bruggeman R, Slooff C, Corpeleijn E, Pijnenborg M. The Effects of Lifestyle Interventions on (Long-Term) Weight Management, Cardiometabolic Risk and Depressive Symptoms in People with Psychotic Disorders: A Meta-Analysis. PloS one. 2014;9(12):e112276.
5. Association of Resistance Exercise, Independent of and Combined With Aerobic Exercise, With the Incidence of Metabolic Syndrome, Mayo Clin Proc. 2017 August ; 92(8): 1214–1222. doi:10.1016/j.mayocp.2017.02.018.
6. Guarner V, Rubio-Ruiz ME. Low-grade systemic inflammation connects aging, metabolic syndrome and cardiovascular disease. Interdiscip Top Gerontol. 2015;40:99–106. https://doi.org/10.1159/000364934.
7. Marsland AL, McCaffery JM, Muldoon MF, Manuck SB. Systemic inflammation and the metabolic syndrome among middle-aged community volunteers. Metab Clin Exp. 2010;59(12):1801–8. <https://doi.org/> 10.1016/j.metabol.2010.05.015.
8. Abdominal obesity and metabolic syndrome: exercise as medicine? Paley and Johnson BMC Sports Science, Medicine and Rehabilitation (2018) 10:7 <https://doi.org/10.1186/s13102-018-0097-1>

**Social Worker Reference**:

1. Heller, T., Roccoforte, J.A., Hsieh, K.’ Cook J.A, & Pickett, S.A (1997). Benefits of support groups for families of adults with severe mental illness. *American Journal of Orthopsychiatry, 67* (2), 187 – 198.

**Clinical Psychologist References:**

1. American Psychological Association. (2022). *Motivational interviewing.* APA Dictionary of Psychology. <https://dictionary.apa.org/motivational-interviewing>
2. Eckel, R. H., Grundy, S. M., & Zimmet, P. Z. (2005). The metabolic syndrome. *The Lancet*, *365*(9468), 1415-1428.
3. Fitch, K. V., Anderson, E. J., Hubbard, J. L., Carpenter, S. J., Wad- dell, W. R., Caliendo, A. M., & Grinspoon, S. K. (2006). Effects of a lifestyle modification program in HIV-infected patients with the metabolic syndrome. *AIDS, 20*, 1843–1850.
4. Toumbourou, J. (2010). Psychological interventions for the prevention and management of lifestyle-related chronic disease. *APS InPsych, 32*(1).
5. Kaur, J. (2014). A comprehensive review on metabolic syndrome. *Cardiology Research and Practice, 2014*, 1-21. <https://doi.org/10.1155/2014/943162>
6. Scott. H. K., Jain, A., & Cogburn, M. (2021). *Behaviour modification.* National Centre for Biotechnology Information. https://www.ncbi.nlm.nih.gov/books/NBK459285/

**Occupational Therapist Reference:**

1. Nielsen S, Christensen J. Occupational Therapy for Adults with Overweight and Obesity: Mapping Interventions Involving Occupational Therapists. Occupational Therapy International. 2018;2018:1-17.

**Pharmacist References:**

1. Lambert T. Managing the metabolic adverse effects of antipsychotic drugs in patient with psychosis. Australian Prescriber. August 2011
2. Woodall A. Managing patients on atypical antipsychotics in primary care. Prescribing in practice. Prescriber November/December 2021
3. Meyer JM, Stahl SM. The clozapine handbook. Cambridge; New York, NY: Cambridge University Press, 2019. First published 2020.
4. Taylor DM, Barnes TRE, Young AH. The Maudsley prescribing guidelines in psychiatry. 13th edition. Hoboken, NJ: Wiley, 2019.
5. The Royal Australian College of General Practitioners. Management of type 2 diabetes: A handbook for general practice. East Melbourne, Vic: RACGP, 2020.
6. Guidelines for the management of absolute cardiovascular disease risk. Endorsed by RACGP 2012.
7. <https://irp.cdn-website.com/5a7b1d9a/files/uploaded/Absolute_CVD_Risk_Full_Guidelines.pdf>

**Medical and Nursing References:**

1. Waterreus A, Laugharne J. Screening for the metabolic syndrome in patients receiving antipsychotic treatment: a proposed algorithm. Medical Journal of Australia. 2009;190(4):185-189.
2. Mark F Harris MBBS, FRACGP, MD, Professor and Director, Centre for Primary Health Care and Equity, University of New South Wales and the Centre for Research Excellence in Obesity Management and Prevention in Primary Health Care, 2020. *RACGP - The Metabolic Syndrome*. [online] Racgp.org.au. Available at: <https://www.racgp.org.au/afp/2013/august/the-metabolic-syndrome> [Accessed 10 June 2020].
3. Royal Australian and New Zealand College of Psychiatrists, 2016. *Clinical Practice Guidelines For The Management Of Schizophrenia And Related Disorders*.
