# Supplementary material for: A streamlined multidisciplinary metabolic clinic in psychiatric recovery service: a pilot study
Source: Front Psychiatry. 2024 Feb 20;15:1344453. doi: 10.3389/fpsyt.2024.1344453 (PMC10913053; doi:10.3389/fpsyt.2024.1344453)
Supplement: Supplementary file 4 [file Table_2.docx]

All patients in Banksia and Waratah Cottages (pilot)

Each cottage has a designated ‘*Metabolic Champion*’

Nursing staff to complete ‘MH Metabolic Monitoring Form’ on eMR **on Rehab admission** and **every 3 months**:

-Systolic and Diastolic BP measurements

-Waist circumference

Nursing staff to start conversations about MetS with patients

*Metabolic Champion* to ensure fasting bloods ordered **every 3 months**, including:

-Fasting HDL

-Fasting Triglycerides

-Fasting blood glucose

*Metabolic Champion* to update blood test on MH Metabolic Monitoring Form

Screening

All consumers in Cottages **A**, **B** and C

Appendix 2. Flow diagram of the Metabolic Clinic

Intervention

Metabolic Focus Group (TBC frequency, duration, content, max ?5 new participants at a time) consists of: SUGGESTIONS BELOW

- risks of MetS (Nursing staff/ Senior Pharmacist/ medical officers)

- operationalising lifestyle changes in the community (Occupational Therapist, Social Worker, DT)

- interactive session on lifestyle recommendations for prevention and management of MetS (DT, Dietician, Ex Phys.)

-Dietician and Ex Phys. will plan their clients’ individual interventions around the Focus Group throughout the week

Social worker and Occupational therapist to facilitate community follow-up and discharge planning, referrals to relevant services in the community, and disability pension liaison for services to improve ongoing metabolic health (e.g. Community Dietitian and Exercise physiologist, Diabetes educator, community programmes, NGOs, etc)

Medical officers to highlight any consumer for risks of MetS on discharge letter to the GP

Exercise physiologist and Dietitian to refer eligible participants to community services (e.g. Get Healthy Service)

The *MetFit Program* is a 12-week structured lifestyle and behavioural change program

Evidence based practice from a multidisciplinary team of: Medical, Clinical Psychology, Pharmacy, Peer workforce, Dietetics, Exercise Physiology and Diversional Therapy

-Education about MetS

-Medication review

-Goal-oriented behavioural changes and long-term sustainable lifestyle habits

-Group exercise activities

-Nutrition education and preparation of healthy meals

Diversional therapist, Dietitian and Exercise physiologist to note down identified consumers; consent to be obtained

Team will invite *all* appropriate consumers in Cottage **A** and **B** who meet the criteria for MetS and/or are at risk of MetS to the *MetFit program*

At the weekly MDT meetings when all the domains in the MH Metabolic Monitoring Form are completed, team to identify:

(Consultant Psychiatrists to oversee)

-metabolic syndrome (>=3 out of 5 to be out of range)

-at risk metabolic syndrome (1-2 out of 5 to be out of range)

Team will invite *all* patients who meet the criteria for MetS and are at risk of MetS to one Metabolic Focus Group.

Identification
