# Supplementary material for: A streamlined multidisciplinary metabolic clinic in psychiatric recovery service: a pilot study
Source: Front Psychiatry. 2024 Feb 20;15:1344453. doi: 10.3389/fpsyt.2024.1344453 (PMC10913053; doi:10.3389/fpsyt.2024.1344453)
Supplement: Supplementary file 5 [file Table_3.docx]

Appendix 3. Cue card on MetS points for discussion

| **Metabolic Syndrome (MetS) points for discussion** |
| --- |
| - The MetS is a cluster of risk factors for heart disease and diabetes |
| - These are - raised fasting plasma glucose, abdominal obesity, raised ‘bad’ cholesterol. ‘low’ good cholesterol and high blood pressure. |
| - The risk for people with MetS for heart disease is **twice** and for type 2 diabetes is **five** fold compared to people without MetS. |
| - Measuring waist Circumference is the initial step to identify MetS; other factors will be checked soon. |
| - Lifestyle change is the best way to prevent increased risk of heart disease and diabetes. |
