# Supplementary material for: A streamlined multidisciplinary metabolic clinic in psychiatric recovery service: a pilot study
Source: Front Psychiatry. 2024 Feb 20;15:1344453. doi: 10.3389/fpsyt.2024.1344453 (PMC10913053; doi:10.3389/fpsyt.2024.1344453)
Supplement: Supplementary file 6 [file Table_4.docx]

Appendix 4. MetFit program

**METFIT**

**12 WEEK Group Program Outline**

| **Week** | **Discipline** | **Activity Focus** | **Overview** | **Measures** |
| --- | --- | --- | --- | --- |
| **1** | **Diversional Therapy** | Introduction to Leisure | - Orientation to the program (meeting the facilitators, format) - Ice breaker: getting to know you - Establishing Group Values - Leisure and recreation education session - Recreation Activity 30 mins - Smoothie - Team photos | - Patients wear pedometers. Steps are recorded after each week. - Baseline leisure and recreation questionnaire - Pre/Post Mood questionnaire - Strengths/Weaknesses/Opportunities/Barriers worksheet - MetFit evaluation form |
|  | **Exercise Physiology** | Data collection | Session 1.  EP to administer:   - Consent form - SMART Goal setting - Whole body stretching - Education and introduction to Exercise Physiology and Metabolic Syndrome   Session 2.   - SMART Goal setting to be completed if not completed in session 1. - 6 minute walk test / submaximal testing - 1 minute push up test - 1 minute squat or sit to stand - Single leg balance | - Mood chart pre/post session - Pre-exercise screening - SIMPAQ - Mood chart pre/post session |
|  | **Dietetics** | Introduction, Goals | - What is MetFit/overview - Metabolic syndrome - Waist circumference, blood sugar levels, blood pressure, cholesterol - Australian Dietary Guidelines - Goal setting | - Pre-nutrition and lifestyle survey - Waist circumference. - Weight, height, BMI. - Bloods (total cholesterol, LDL, HDL, triglycerides, B12, folate, Vitamin D). |
| **2** | **Diversional Therapy** | Goal Setting | - Education on SMART goals - Consumers to identify a goal on the worksheet - Recreation Activity 30 mins | - Steps - Pre/Post Mood questionnaire |
|  | **Exercise Physiology** | Commence structured exercise program. | Session 3.  Commence structured exercise program.   - Discuss form and technique. Introduction to exercises and muscle groups. - Dynamic warm up 5 mins - Cardio machines 20 mins (2 machines) - Resistance machines/weights/body weight 20 mins - Whole body static stretches 5 mins   Session 4.   - Dynamic warm up 5 mins - Cardio machines 20 mins (2 machines) - Resistance machines/weights/bodyweight 20 mins - Whole body static stretches 5 mins | Mood chart pre/post session |
|  | **Dietetics** | The Australia Guide to Healthy Eating and the Five Food groups | - The Australia Guide to Healthy Eating - The Five Food groups - Food and beverage diary - Health eating activity   Cooking: Smashed avocado and egg |  |
| **3** | **Diversional Therapy** | Different types of leisure/Practical component | - Review and discuss the SMART goal from the worksheet. - Consumers to develop their action plan (refer to the goal setting worksheet). - Facilitators sit with each person to discuss how to get started - Recreation Activity: 6 station fitness circuit 30 mins - Smoothie | - Steps - Pre/Post Mood questionnaire |
|  | **Exercise Physiology** |  | Session 5 and 6.  AS PER WEEK 2 PLAN  Implement progressive overload principles.   - EP to review exercise goals, exercise program and modify where required. |  |
|  | **Dietetics** | Cholesterol and types of fat | - Cholesterol types (LDL, HDL, Trans) - Types of dietary fats (unsaturated, saturated, trans fats). - Healthy takeaway swaps - Teaspoons in fat in takeaway foods |  |
| **4** | **Diversional Therapy** | Activate | - Recreation Activity. Minute to win it. 45 mins | - Steps - Pre/Post Mood questionnaire |
|  | **Exercise Physiology** |  | Session 7 and 8.  AS PER WEEK 2 PLAN  Implement progressive overload principles.  EP to review exercise goals, exercise program and modify where required. |  |
|  | **Dietetics** | Portion control and how to build a balanced meal | - Portion control and building a balanced meal - Portions using hand cues - Protein foods - Carbohydrate and low GI foods - Healthy fats - Flavour boosters - Activity with food models and portion plates | - Waist circumference. - Weight, height, BMI. |
| **5** | **Diversional Therapy** | Activate | - Recreation Activity: Parramatta Lake Hike 45 mins - Smoothie | - Steps - Pre/Post Mood questionnaire |
|  | **Exercise Physiology** |  | Session 9 and 10.  AS PER WEEK 2 PLAN  Implement progressive overload principles.  EP to review exercise goals, exercise program and modify where required. |  |
|  | **Dietetics** | Sugar and ways to reduce intake | - High sugar foods and beverages - Amount of added sugar in common beverages - Importance of drinking water and strategies to increase water intake |  |
| **6** | **Diversional Therapy** | Halfway | - Consumers to review their goal from week 2 to make sure they are on the right track. - Affirmation activity - Recreation Activity 45 mins | - Steps - Pre/Post Mood questionnaire - MetFit evaluation form |
|  | **Exercise Physiology** |  | Session 11 and 12.  AS PER WEEK 2 PLAN  Implement progressive overload principles.  EP to review exercise goals, exercise program and modify where required. |  |
|  | **Dietetics** | Mindful Eating | - Hunger scale - Physical Vs psychological hunger - Mindfulness activity - Activity with healthy snacks |  |
| **7** | **Diversional Therapy** | Activate | - Recreation Activity. 6 station fitness circuit (do it twice). 45 mins - Smoothie | - Steps - Pre/Post Mood questionnaire |
|  | **Exercise Physiology** |  | Session 13 and 14.  AS PER WEEK 2 PLAN  Implement progressive overload principles.  EP to review exercise goals, exercise program and modify where required. |  |
|  | **Dietetics** | Salt and blood pressure | - How salt can increase blood pressure - Identify high salt foods - Strategies to reduce salt intake   Cooking: Tuna and vegetable pasta |  |
| **8** | **Diversional Therapy** | Activate | - Recreation Activity. Minute to win it. 45 mins | - Steps - Pre/Post Mood questionnaire |
|  | **Exercise Physiology** |  | Session 15 and 16.  AS PER WEEK 2 PLAN  Implement progressive overload principles.  EP to review exercise goals, exercise program and modify where required. |  |
|  | **Dietetics** | Label reading | - Ingredient lists - Reading food labels - Health claims | - Waist circumference. - Weight, height, BMI. |
| **9** | **Diversional Therapy** | Activate in the community | - Recreation Activity (aqua aerobics/hydrotherapy/bike riding/indoor rock climbing) 60 mins | - Steps - Pre/Post Mood questionnaire |
|  | **Exercise Physiology** |  | Session 17 and 18.  AS PER WEEK 2 PLAN  Implement progressive overload principles.  EP to review exercise goals, exercise program and modify where required. |  |
|  | **Dietetics** | Healthy snacks | - How to identify a healthy snack - Importance of regular meal pattern - High/low GI   Cooking: healthy snacks |  |
| **10** | **Diversional Therapy** | Activate in the community | - Recreation Activity (Scavenger hunt) 60 mins | - Steps  - Pre/Post Mood questionnaire |
|  | **Exercise Physiology** |  | Session 19 and 20.  AS PER WEEK 2 PLAN  Implement progressive overload principles.  EP to review exercise goals, exercise program and modify where required. |  |
|  | **Dietetics** | Meal Planning | - How and why to plan meals and snacks |  |
| **11** | **Diversional Therapy** | Activate in the community | - Recreation Activity (consumers choice) 60 mins | - Steps - Pre/Post Mood questionnaire |
|  | **Exercise Physiology** |  | Session 19 and 20.  AS PER WEEK 2 PLAN  Implement progressive overload principles.  EP to review exercise goals, exercise program and modify where required. |  |
|  | **Dietetics** | Budgeting and supermarket shopping tips | - Food grocery shopping list - Budget tips   Cooking: Meal suggested by participants |  |
| **12** | **Diversional Therapy** | Completion | - Recreation Activity 30 mins - Review goals - Group discussion (self-reflection, share a positive story about your time in the programs) - Sharing affirmations - Steps competition results - Smoothie | - Steps - Pre/Post Mood questionnaire - MetFit evaluation form |
|  | **Exercise Physiology** | Functional testing | - 6MWT / submaximal testing - 1 minute push up test - 1 minute squat or STS - Balance (SLB) - Flexibility (Hamstring) - Complete SIMPAQ | - Mood chart pre/post session |
|  | **Dietetics** | Smart food swaps – recap week | - Food swaps to reduce sugar, saturated fat, salt intake. | - Post-nutrition and lifestyle survey - Waist circumference. - Weight, height, BMI. - Bloods (total cholesterol, LDL, HDL, triglycerides, B12, folate, Vitamin D). |
| **13** | **All clinicians** | Graduation ceremony and celebration lunch for project participants |  |  |
