# Supplementary material for: A streamlined multidisciplinary metabolic clinic in psychiatric recovery service: a pilot study
Source: Front Psychiatry. 2024 Feb 20;15:1344453. doi: 10.3389/fpsyt.2024.1344453 (PMC10913053; doi:10.3389/fpsyt.2024.1344453)
Supplement: Supplementary file 7 [file Table_5.docx]

**EVALUATION FORM**

**Program: MetFit Date: _________________**

*Please mark each criteria out of 5 using the following scale:* ***(Please Circle)***

**Poor**  **Average**  **Good**  **Very Good**   **Excellent**

**1**  **2**  **3**  **4**  **5**

1. **The program helped me understand the importance of exercise and identify ways to exercise safely.**

**1 2 3 4 5**

1. **The program helped me understand the importance of healthy eating and identify ways to eat well.**

**1**  **2**  **3**  **4**  **5**

1. **I feel confident about looking after my health and wellbeing.**

**1**  **2**  **3**  **4**  **5**

1. **This program increased my ability to participate in meaningful leisure and recreation activities.**

**1**  **2**  **3**  **4**  **5**

1. **I am proud of myself and of my achievements.**

**1 2 3 4 5**

1. **How would you rate the program?**

**1 2 3 4 5**

1. **What did you like or dislike about the program?**

**___________________________________________________________**
